# Modulation of post-stroke degenerative and regenerative processes and subacute protection by site-targeted inhibition of the alternative pathway of complement

**DOI:** 10.1186/s12974-015-0464-8

**Published:** 2015-12-30

**Authors:** Ali Alawieh, Andrew Elvington, Hong Zhu, Jin Yu, Mark S. Kindy, Carl Atkinson, Stephen Tomlinson

**Affiliations:** Department of Microbiology and Immunology, Children’s Research Institute, Medical University of South Carolina, 173 Ashley Avenue BSB 201, Charleston, SC 29425 USA; Department of Neuroscience, Neuroscience Institute, Medical University of South Carolina, Charleston, SC USA; Ralph H. Johnson Veteran Affairs Medical Center, Charleston, SC USA

**Keywords:** Ischemic stroke, Complement inhibition, Neuroinflammation, Neuroprotection

## Abstract

**Background:**

Complement promotes neuroinflammation and injury in models of stroke. However, complement is also being increasingly implicated in repair and regeneration after central nervous system (CNS) injury, and some complement deficiencies have been shown to provide acute, but not subacute, protection after murine stroke. Here, we investigate the dual role of complement in injury and repair after cerebral ischemia and reperfusion.

**Methods:**

We used complement-deficient mice and different complement inhibitors in a model of transient middle cerebral artery occlusion to investigate complement-dependent cellular and molecular changes that occur through the subacute phase after stroke.

**Results:**

C3 deficiency and site-targeted complement inhibition with either CR2-Crry (inhibits all pathways) or CR2-fH (inhibits alternative pathway) significantly reduced infarct size, reduced apoptotic cell death, and improved neurological deficit score in the acute phase after stroke. However, only in CR2-fH-treated mice was there sustained protection with no evolution of injury in the subacute phase. Whereas both inhibitors significantly reduced microglia/macrophage activation and astrogliosis in the subacute phase, only CR2-fH improved neurological deficit and locomotor function, maintained neurogenesis markers, enhanced neuronal migration, and increased VEGF expression. These findings in CR2-fH-treated mice correlated with improved performance in spatial learning and passive avoidance tasks. The complement anaphylatoxins have been implicated in repair and regenerative mechanisms after CNS injury, and in this context CR2-fH significantly reduced, but did not eliminate the generation of C5a within the brain, unlike CR2-Crry that completely blocked C5a generation. Gene expression profiling revealed that CR2-fH treatment downregulated genes associated with apoptosis, TGFβ signaling, and neutrophil activation, and decreased neutrophil infiltration was confirmed by immunohistochemistry. CR2-fH upregulated genes for neural growth factor and mediators of neurogenesis and neuronal migration. Live animal imaging demonstrated that following intravenous injection, CR2-fH targeted specifically to the post-ischemic brain, with a tissue half-life of 48.5 h. Finally, unlike C3 deficiency, targeted complement inhibition did not increase susceptibility to lethal post-stroke infection, an important consideration for stroke patients.

**Conclusions:**

Ischemic brain tissue-targeted and selective inhibition of alternative complement pathway provide self-limiting inhibition of complement activation and reduces acute injury while maintaining complement-dependent recovery mechanisms into the subacute phase after stroke.

**Electronic supplementary material:**

The online version of this article (doi:10.1186/s12974-015-0464-8) contains supplementary material, which is available to authorized users.

## Background

Following onset of cerebral ischemia, many stroke patients show reperfusion of their infarct either spontaneously or as a secondary effect of thrombolytic therapy. Cerebral reperfusion initiates a cascade of pathophysiological events that cause secondary injury, which can lead to greater tissue damage and more severe functional and cognitive deficits. Clinical observations and experimental studies indicate a central role for complement in the propagation of ischemia reperfusion injury (IRI) in both the central nervous system (CNS) and in non-CNS tissue [1, 2].

Cumulative evidence indicates that following cerebral ischemia and reperfusion, complement is activated via the lectin pathway and amplified via the alternative pathway (reviewed in [3]). Deficiency or pharmacologic inhibition of either pathway is protective in the acute phase following murine ischemic stroke [4–8]. However, from a clinical standpoint, it is important to determine the effect of any potential therapeutic strategy on longer-term outcome after stroke. While complement activation may be injurious in the acute phase, there is evidence that complement also has neuroprotective functions in the CNS, including after stroke [9, 10]. In this regard, it has been shown that the protective effect of lectin pathway deficiency is not sustained in the subacute phase of stroke [11], even though there is a wide therapeutic window for lectin pathway inhibition and acute protection [5]. In addition, deficiency in C3, a central protein of all complement pathways, provides effective protection in the acute phase [12, 13], but not the subacute phase following ischemic stroke [14].

In a previous study, we demonstrated that alternative pathway-deficient (fB−/−) and inhibited (CR2-fH treated)-mice are protected from acute injury after cerebral ischemia and reperfusion. We further demonstrated that alternative pathway inhibition resulted in reduced infarct volumes and reduced neurological deficit scores in the subacute phase (7 days) after ischemia [4]. In the current study, we follow up on this preliminary observation and investigate how complement modulates the cellular and molecular events that contribute to post-stroke degenerative and regenerative processes, and how this relates to cognitive impairment and functional recovery. We also investigate whether subacute protection is a specific property of the alternative pathway. For this study, we utilize CR2-fH, an inhibitor shown to specifically inhibit the alternative pathway [15], and CR2-Crry, an inhibitor of all complement pathways that has been shown to provide acute protection after stroke, but that has not been investigated with regard to subacute outcome [13, 16]. The complement inhibitors Crry and fH are targeted to sites of complement activation (C3d deposition) via the complement receptor 2 (CR2) moiety [16].

## Methods

### CR2 fusion proteins

The complement inhibitors CR2-Crry and CR2-fH were constructed, expressed, and purified as previously described [15, 16]. For quality control, complement inhibitory activity was tested by Zymosan assay as described [17].

### Transient ischemic stroke model

Two cohorts of male 8- to 10-week-old wt C57BL/6 mice or C3−/− mice on C57BL/6 background (Jackson Laboratories) were independently subjected to transient ischemic stroke, with 60-min middle cerebral artery occlusion (MCAO) [13]. In the first cohort, wt mice received one treatment of 0.25 mg CR2-Crry, 0.4 mg CR2-fH, or 100 μl phosphate buffered saline (PBS) vehicle given intravenously 30 min following reperfusion. Dosing was based on efficacy titrations performed in a model of intestinal IRI [15, 16]. Following either 24 h or 7 days of reperfusion, animals were sacrificed and brains processed for histology or protein extraction. For antibiotic prophylaxis, C3−/− mice received 250 mg/L ciprofloxacin (VetSource) in drinking water ad libitum 14 days prior to MCAO and until sacrifice. In the second cohort, wt mice received either one treatment of 0.4 mg CR2-fH or 100 μl PBS vehicle 30 min following reperfusion. Following either 3 or 7 days of reperfusion, mice were assessed for neurological outcome as well as performance on Barnes maze and passive avoidance tests. Mice were sacrificed on day 7 and brains processed for histology or protein extraction. Cerebral blood flow was assessed by Laser Doppler Flowmetry (Moor Instruments) before, during, and following ischemia; mice were excluded if not achieving a reduction in blood flow to 20 % of pre-ischemia levels. Temperature, blood pressure, and heart rate were also monitored before, during, and following ischemia, and consistent with previous reports, no differences were observed [13]. All groups were randomly assigned, and experimenters were blinded to identity of groups. All experiments and procedures were conducted in accordance with the *Guide for the Care and Use of Laboratory Animals* by the National Institutes of Health and approved by the Institutional Animal Care and Use Committee at the Medical University of South Carolina.

### Neurological outcomes

Neurological deficit was assessed following MCAO by a blinded observer using a five-point scoring system, as previously described [18]. Additionally, locomotor activity was automatically quantified using the Versamax open field activity monitor (AccuScan Instruments). Mice were placed in a random corner and allowed to acclimate for 10 min prior to a 60-min testing period. External noise, lights, and other stimuli were minimized to reduce bias. Several measures were automatically retrieved during the task including total distance moved, number of movements, time spent at periphery, and time spent at the center. Activity readings taken prior to sham procedure were used to establish any differences in baseline activities. The duration that the animal spent at the periphery vs. the center was used to assess anxiety level during the task.

### Behavioral testing

Animals of the second cohort were tested for their performance on both Barnes maze and passive avoidance tasks. To assess spatial reference memory, mice were trained on Barnes maze for 5 days before surgery, as previously described [19], then tested again on days 3 and 7 after reperfusion for the time needed to escape into the hole, the number of error pokes, and the length of the animal’s path prior to escape. An automated passive avoidance apparatus (Coulbourn Instruments) was used to assess avoidance learning with automated sensing and shock systems (GraphicState® 4, Coulbourn Instruments). The apparatus included a double compartment chamber with one lit and one dark compartment. Mice were allowed to explore the chamber for 5 min on habituation phase. Following habituation, the mice were given one trial where a shock is associated with the dark side, allowed 48 h of rest, and then tested for retention measured as latency to enter the dark side. Testing was repeated on days 3 and 7 post-reperfusion with no shock delivered during test phase.

### Histological and immunohistochemical analysis

For analysis of infarct volume, brains were perfused transcardially with PBS before their removal, then cut into 2-mm coronal sections and stained with 2 % triphenyltetrazolium chloride (TTC) [20]. Infarct volumes were analyzed with ImageJ software (National Institutes of Health) and calculated as percentage of total brain from summation of four sections obtained 2 mm apart. Immunohistochemical staining was conducted on 8-μm paraffin sections and assessed by a blinded observer by light microscopy (Olympus BX61). Following antigen retrieval (IHC World), the following primary antibodies were used: anti-ionized calcium-binding adaptor molecule 1 (Iba-1, 1:250; Abcam), anti-mouse glial fibrillary acidic protein (GFAP, 1:1000; Dako), anti-von Willibrand Factor (vWF, 1:500; Dako), anti-Ki67 (1:500; Abcam), anti-doublecortin (Dcx, 1:200; Millipore), and anti-Gr-1 (1:100, Stem Cell). Primary antibodies were detected with ImmPress-HRP kit and NovaRed peroxidase chromagen (Vector Laboratories), and primary antibodies were omitted for negative controls. Terminal deoxynucleotidyl transferase dUTP nick end labeling (TUNEL) staining was performed using ApopTag Peroxidase Staining Kit (Millipore) per manufacturer’s instructions. For birth dating proliferating neuroblasts, intraperitoneal BrdU was administered to a subset of mice every other day starting 24 h after reperfusion, and BrdU-positive cells were detected by anti-BrdU antibody (1:300; Sigma).

### Quantification of staining

Paraffin sections were obtained +2 to −2 mm relative to bregma, and sections 200 μm apart were used for quantification. The ischemic core and the penumbra (area surrounding the ischemic core) were confirmed with Luxol fast blue staining or counterstaining, and random fields were examined within the penumbra using an automated motorized stage on an Olympus BX61 using Visiopharm software. Counting of apoptotic cells was performed on an average of eight sections per brain, with the entire ipsilateral hemisphere being quantified, and density was calculated as number of positive cells per square millimeter of tissue. Reactive astrocytes, microglia/macrophages, and vWF positive vessels were quantified on sections 200 μm apart. An average of five sections/brain was quantified. Cells were counted in randomly generated fields, and density was calculated as number of positive cells per square millimeter of tissue. Dcx- and Ki67-positive cells were quantified within the subventricular zone (SVZ) of the lateral ventricles, the subgranular zone (SGZ) of the dentate gyrus, the basal ganglia, and the hippocampus from ×40 magnification random fields on sections 25 to 50 μm apart (three sections/brain). All analyses were done by light microscopy (Olympus BX61 with Visiopharm image acquisition software) by an observer blinded to the experimental groups.

### Tissue protein analysis

Following sacrifice, brains were divided into ipsilateral and contralateral hemispheres, and protein was extracted by homogenization in NP-40 lysis buffer (Invitrogen) containing 1 mm PMSF (Sigma-Aldrich), 92.6 μm FUT175 (BD Biosciences) and 5 μL of protease inhibitor cocktail (Sigma-Aldrich). Protein concentrations were determined using BCA protein assay kit (Thermo Scientific). Vascular endothelial growth factor (VEGF; Abcam), complement C5a (BD Biosciences), and β-actin (Sigma-Aldrich) were assessed through Western blotting of 25 μg total protein, with SDS-PAGE run under reducing conditions, and detected with HRP-conjugated secondary antibodies (Vector Laboratories). Signal densities were calculated relative to β-actin and normalized to wt groups.

### Nanostring analysis

Eight C57BL/6 mice (8 weeks of age) were also subjected to 1 h MCAO and treated with either CR2-fH or PBS vehicle (*n* = 4/group) to assess the effect of CR2-fH on gene expression profile after MCAO. Animals were perfused with PBS 72 h after reperfusion, and their brains were extracted for RNA Extraction. Gene expression analysis was performed using the Nanostring nCounter Analysis System (Nanostring Technologies) using a custom-designed codeset containing 249 genes involved in immunological and cell survival processes (Additional file 1) [21]. Each reaction contained 250 ng of total RNA in a 5-μL aliquot, plus reporter and capture probes, and six pairs of positive control and eight pairs of negative control probes. Analysis and normalization of the raw Nanostring data was conducted using nSolver Analysis Software v1.1 (Nanostring Technologies). Raw counts were normalized to levels of reference gene. A background count level was estimated using the average count of the eight negative control probes in every reaction plus two SDs.

### Animal imaging

Five C57BL/6 mice (8 weeks of age) had their heads shaved and treated with Nair 2 days prior to MCAO surgery described above. Fluorescently labeled CR2-fH was injected according to the therapeutic protocol described above. CR2-fH was labeled using Xenolight C750 NIR Fluorescent Dye according to manufacturers instructions (Perkin Elmer). Mice were anesthetized and imaged at 6, 24, 48, 72 and 96 h and at 7 days post-injection using a Maestro EX imaging system (Perkin Elmer). Sham-operated animals were also injected as controls. Fluorescent signal was quantified with supplied software, and tissue half-life was calculated according to the formula *t*_1/2_ = *t* × ln(2)/ln(*N*_0_/*N*_t_), where *t* = fluorescence signal after time gone by, *N*_0_ = signal at beginning (6 h), *N*_t_ = signal after period of time (90 h).

### Statistics

Statistical differences between parametric data (infarct volumes, activity values, ELISA values, cell counts, densitometry, and Nanostring data) were assessed using one-way analysis of variance (ANOVA) test with Bonferroni’s multi-group comparison, and non-parametric data (neurological deficits) were compared with the Kruskal-Wallis test with Dunn’s comparison (Prism 5.0, GraphPad). Survival was compared from all mice subjected to MCAO using the Kaplan-Meier test. Differences between data were considered statistically significant when *p* < 0.05.

## Results

### Targeted complement inhibition reduces the extent of injury post-MCAO

**Fig. 1:**
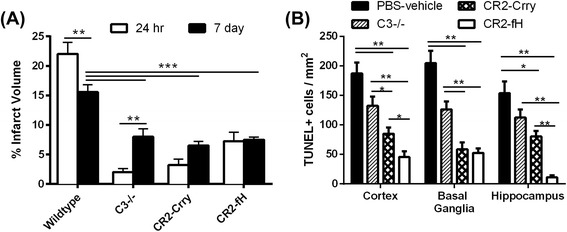
Complement inhibition reduces cerebral injury and cell death following MCAO and 7 days reperfusion. **a** Infarct volumes after 60 min MCAO and either 24-h or 7 days reperfusion. Mean +/− SEM, *n* = 8–12 mice. **p < 0.01, ***p < 0.001, #*p* < 0.001. **b** Cell death in different brain regions as analyzed by TUNEL immunostaining at 7 days after MCAO. Mean +/− SEM (TUNEL-positive cells per square millimeter), *n* = 5–6 mice. *p < 0.05

It has been shown previously that C3 deficiency [12, 13] or treatment of wt mice with CR2-Crry [16] or CR2-fH [15] significantly reduces infarct volume 24 h after MCAO and reperfusion. Here, we investigated the effect of targeted complement inhibition on subacute injury by assessing infarct volumes 7 days after MCAO, and compared subacute with acute (24 h) outcomes. Pharmacological inhibition in all studies reported here was achieved with a single dose of inhibitor (CR2-Crry or CR2-fH) administered 90 min after ischemia and 30 min after reperfusion. Compared to wt control mice, infarct volume was significantly decreased in C3-deficient mice and in wt mice treated with either CR2-Crry or CR2-fH at both 24 h and 7 days post-MCAO (Fig. 1a). At 24 h after MCAO, there was no significant difference in infarct volume between C3-deficient mice and CR2-fH- or CR2-Crry-treated mice. However, unlike CR2-fH-treated mice (and wt controls), C3-deficient and CR2-Crry-treated mice had a rebound increase in infarct volumes at 7 days compared to 24 h post-MCAO. CR2-fH-treated mice did not show any significant difference in infarct volume between the two time points post-reperfusion. We also assessed cell death in the ipsilateral cortex, basal ganglia, and hippocampus 7 days following MCAO using TUNEL staining. CR2-Crry- and CR2-fH-treated mice showed a significant reduction in cell death in the basal ganglia and cortex compared to C3−/− and PBS-treated mice, but only CR2-fH-treated mice had a significant reduction in hippocampal cell death compared to all other groups. Further, compared to CR2-Crry-treated mice, CR2-fH-treated mice had significantly less cell death in the cortex and hippocampus. There were no significant differences between C3−/− mice and control mice across the three brain regions (Fig. 1b). Thus, there is an evolution of secondary injury subacutely after MCAO in C3-deficient mice and CR2-Crry-treated mice, but not in CR2-fH-treated mice.

### Targeted complement inhibition reduces subacute microglia/macrophage activation and astrogliosis after MCAO

**Fig. 2:**
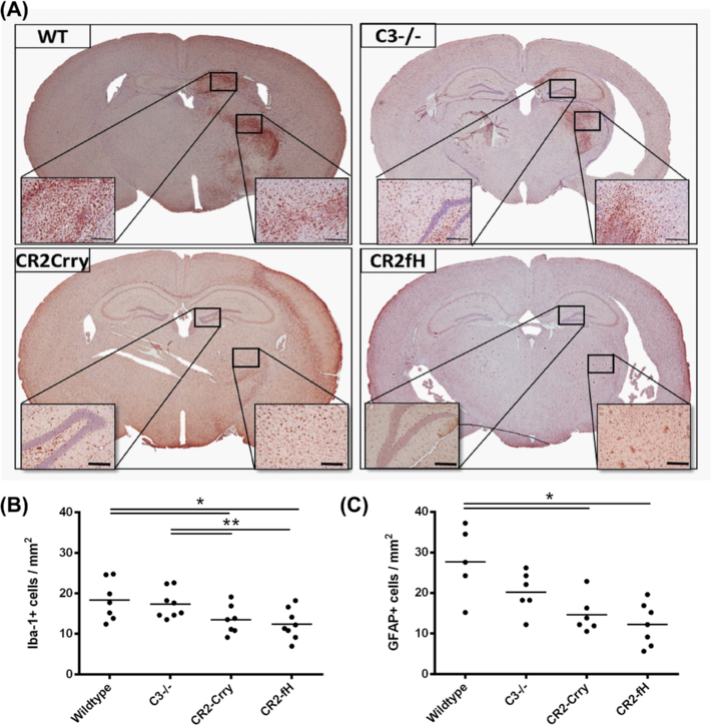
Complement inhibition reduces microglia/macrophage activation and astrogliosis following MCAO and 7 days reperfusion. **a** Representative images of activated microglia/macrophages as assessed by Iba-1 immunohistochemical detection across the different groups. Scale bar = 200 μm. **b** Quantification of Iba-1 immunohistochemical staining. Mean +/− SEM (positive cells per square millimeter), *n* = 6–8 mice. *p < 0.05, **p < 0.01. **c** Quantification of reactive astrocytes as assessed by GFAP immunohistochemical detection. Mean +/− SEM (positive cells per square millimeter), *n* = 5–7 mice. *p < 0.05

Microglia and astrocytes become activated in response to pathological changes, and microglia/macrophage activation and astrogliosis mark inflammation within the CNS, which is reduced at 24 h after MCAO by complement inhibition [4]. Microglia/macrophage activation and astrogliosis in the subacute phase at 7 days post-MCAO was assessed by immunohistochemical detection of Iba-1 and GFAP markers in the striatum, respectively. Compared to wt controls, there was a significant reduction in both activated microglia/macrophages and reactive astrocytes in CR2-Crry- and CR2-fH-treated mice (Fig. 2). In contrast, there was no significant difference in the detection of either marker in C3-deficient mice compared to wt. No differences were observed in the contralateral hemispheres between groups. These data indicate that C3 inhibition, but not C3 deficiency, reduce glial cell activation in the subacute phase after MCAO.

### Effect of complement inhibition on neurological outcome and locomotor function after MCAO

**Fig. 3:**
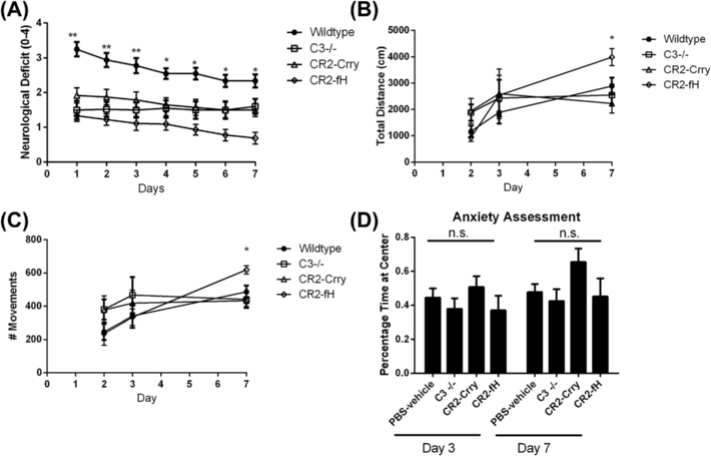
Effect of complement inhibition on behavioral deficit and locomotor function after MCAO. **a** Neurological deficit scores (0–4) within the first week after MCAO. Mean +/− SEM, *n* = 8–13. One-way ANOVA, **p < 0.05 (wild-type compared to all other groups), *p < 0.05 (wild-type compared to CR2-fH only). **b** Anxiety assessment across the different groups measured as percent time spent at the center of an open field. Mean +/− SEM. (One-way ANOVA, n.s. *p* > 0.05). **c**, **d** Locomotor activity measured as total distance moved **(c)** or number of movements **(d)**, using an open field activity monitor. Determinations were made 2, 3, and 7 days after MCAO. Mean +/− SEM, *n* = 8–13. One-way ANOVA, *p < 0.05(CR2-fH-treated compared to wild-type)

We next determined whether the reduced injury and inflammation seen in complement-inhibited mice translated into improvement in neurological outcome after MCAO. Both CR2-Crry and CR2-fH have been shown to improve neurological deficits 24 h after MCAO [4, 13], and here, we used the same deficit scoring method to assess neurological outcome over a 1-week period post-MCAO. Compared to wt controls, C3 deficiency and treatment with either CR2-Crry or CR2-fH significantly improved neurological deficit scores up to 72 h post-MCAO (Fig. 3a). However, only CR2-fH-treated mice continued to display significantly improved neurological deficit scores at 7 days post-MCAO.

To more objectively assess neurological deficit, we measured locomotor activity using an open field activity monitor. There was no significant difference in locomotor activity between any group up to 3 days post-MCAO, but on day 7, CR2-fH-treated mice had significantly increased locomotor activity compared to all other groups (Fig. 3b, c). Since a difference in anxiety levels may confound performance on open field as well as other tasks, we also assessed anxiety levels across the groups by measuring the percentage of time spent at the center of an open field. We found no significant differences in anxiety levels among the different groups on days 3 and 7 post-MCAO (Fig. 3d). The picture emerging from the data thus far indicates that in the subacute phase after stroke, CR2-fH provides better protection than CR2-Crry or C3−/− in terms of evolution of secondary injury and neurological outcome.

### CR2-fH maintains neurogenesis and enhances neuronal migration in the subacute phase after MCAO

**Fig. 4:**
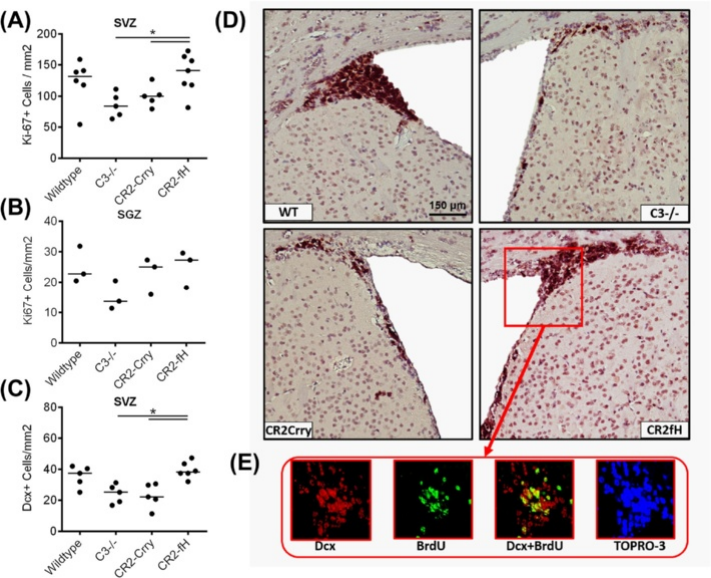
CR2-fH increases subventricular zone neuronal proliferation and neuroblast presence at 7 days post-MCAO. **a** Doublecortin (Dcx^+^) neuroblasts within the ipsilateral SVZ at 7 days post-MCAO. *Bar* = Mean, *n* = 5–7. **b** Ki-67^+^ proliferating cells within the ipsilateral subventricular zone (SVZ) of the lateral ventricles. *Bar* = Mean. **c**, the subgranular zone (SGZ) of the dentate gyrus. *Bar* = Mean (density calculated from five random HPF), *n* = 3–7. *p < 0.05. **d** Representative images of immunostained sections, ×20 magnification. Scale bar = 150 μm. **e** Representative immunofluorescence double staining of SVG in CR2-fH-treated mice with anti-BrdU (*green*) and anti-Dcx (*red*), showing co-localization of signal

**Fig. 5:**
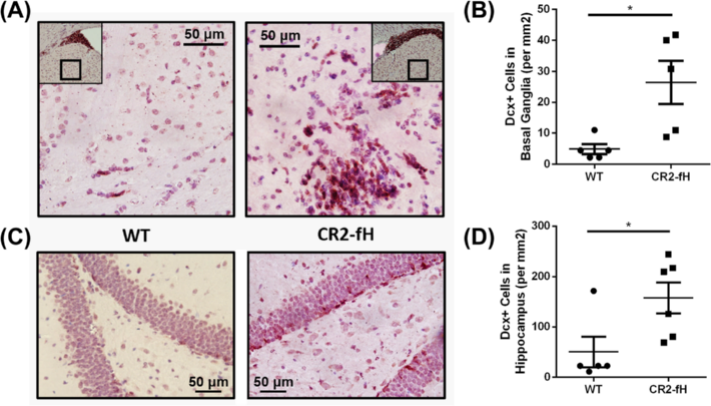
CR2-fH increases neuroblasts migration to perilesional basal ganglia and hippocampus at 7 days post-MCAO. **a** Representative images of neuroblast migration to the basal ganglia. **b** Quantification of number of Dcx + cells in basal ganglia. *Bar* = Mean +/− SEM, *n* = 5. *p < 0.05. **c** Representative images of neuroblasts in the hippocampus. **d** Quantification of number of Dcx + cells in hippocampus. *Bar* = Mean +/− SEM, *n* = 5–6. *p < 0.05

Since complement activation products have been implicated in ischemia-induced neurogenesis that contributes to recovery, we investigated the effect of complement inhibition on proliferation and neurogenesis in the brain after MCAO. Immunostaining for the proliferation marker Ki-67 was performed on sections from the subventricular zone (SVZ) of the lateral ventricles and the subgranular zone (SGZ) of the dentate gyrus, neurogenic niches of the adult brain previously studied in the context of ischemia-induced neurogenesis [22–24]. C3-deficient and CR2-Crry-treated mice, but not CR2-fH-treated mice, had a significant reduction in numbers of proliferating cells within the ipsilateral SVZ compared to wt controls, indicating that, unlike total complement blockade, alternative pathway blockade does not inhibit ischemia-induced neurogenesis (Fig. 4a). No differences were observed between any group in the ipsilateral SGZ (Fig. 4b), or in contralateral regions (data not shown). Immunostaining for doublecortin (Dcx), another marker of neurogenesis that is expressed by immature neurons (neuroblasts), similarly showed a higher number of Dcx-positive cells in the ipsilateral SVZ of CR2-fH-treated mice compared to CR2-Crry-treated mice or C3-deficient mice (Fig. 4c, d). No differences in numbers of Dcx-positive cells were observed between any group in the contralateral SVZ (data not shown). Since CR2-fH treatment did not significantly increase neurogenesis compared to wt mice at 7 days post-MCAO, we further investigated a potential effect of CR2-fH on neuronal migration by quantifying the number of Dcx-positive cells in the ipsilateral basal ganglia and hippocampus of mice post-MCAO; CR2-fH treatment significantly increased neuroblastic cell migration from the SVG to the basal ganglia (Fig. 5a, b) and hippocampus at 7 days after injury (Fig. 5c, d).

### CR2-fH less effectively blocks complement activation than CR2-Crry

**Fig. 6:**
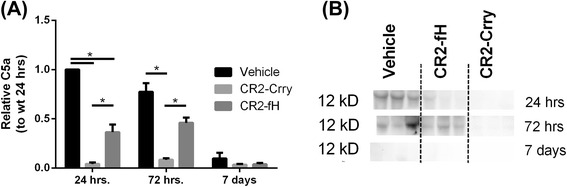
Different levels of C5a generation in brains from CR2-fH- and CR2-Crry-treated mice through subacute phase after stroke. **a** Relative C5a expression as determined by density scan of Western blot of ipsilateral brain tissue extract. Shown are relative expression levels normalized to level in wt control mice at 24 h. Mean +/− SEM, *n* = 4 animals per group, *p < 0.05. **b** Representative Western blot for C5a in brain homogenate

Complement activation products, specifically C3a and/or C5a, have been implicated in promoting neuroregeneration after CNS injury. Therefore, since CR2-Crry inhibits all complement pathways and CR2-fH inhibits only the alternative pathway, a quantitative difference in the level of complement activation is one potential mechanism that could account for the different subacute outcomes and difference in SVZ neurogenesis. To assess this, we measured C5a levels in the ipsilateral hemisphere of mice on days 1, 3, and 7 after MCAO (Fig. 6). Compared to controls, CR2-fH significantly inhibited C5a generation when measured at 1 and 3 days after MCAO, but to a lesser extent than CR2-Crry, which almost completely blocked C5a generation. No differences in C5a levels were detected across the groups on day 7. This result is consistent with CR2-fH providing sufficient complement inhibition to provide protection from injury in the acute phase, while allowing for lectin and/or classical pathway generation of complement activation products at a level sufficient for promoting recovery and neurogenesis in the subacute phase.

### Profiling of gene expression changes after CR2-fH treatment.

**Fig. 7:**
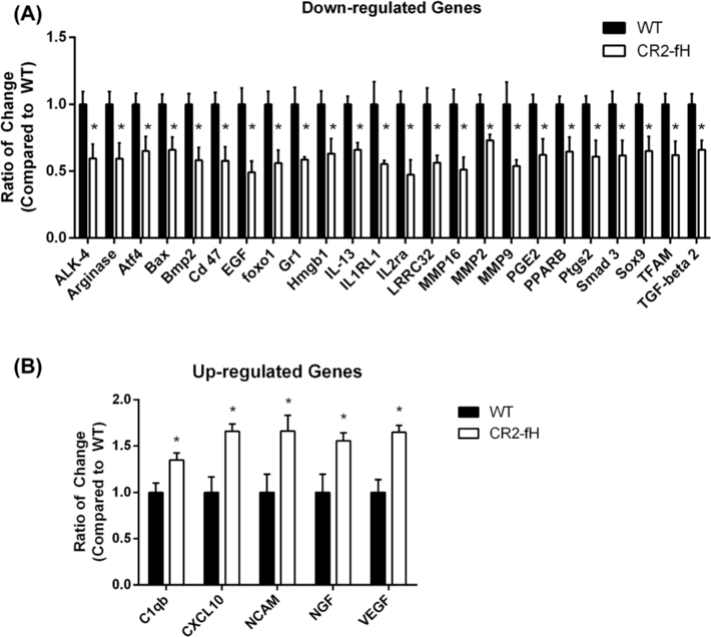
Nanostring analysis of changes in mRNA expression profile secondary to CR2-fH treatment at 7 days post-MCAO. **a** Genes with significantly reduced expression after CR2-fH treatment that exhibit more than 0.8-fold reduction in expression levels. Levels are normalized to the Mean of the wt controls. Mean +/− SEM, *n* = 4, *p < 0.05. **b** Genes with significantly increased expression after CR2-fH treatment that exhibit more than 1.25-fold reduction in expression levels. Levels are normalized to the Mean of the wt controls. Mean +/− SEM, *n* = 4, *p < 0.05. A complete dataset of different analyzed transcripts is provided in Additional file 1

Due to the improved subacute outcomes observed with CR2-fH treatment compared to CR2-Crry treatment of C3 deficiency, we further investigated the effect of CR2-fH treatment by analysis of gene expression profiles 3 days after MCAO. We used Nanostring analysis for high-sensitive capture of mRNA transcripts among a panel of 248 genes involved in immunological, inflammatory, and cell death processes (see Additional file 1 for full list). Among the identified hits of genes exhibiting a significant difference between CR2-fH-treated and wt groups (Additional file 1), genes whose expression showed more than 25 % increase or decrease are shown in Fig. 7a, b. Apoptotic markers (Bax, Atf4), components of TGFβ signaling (TGFβ 2, BMP2, Smad3), and markers of neutrophil activation (Gr-1, MMP9) were among the downregulated genes, which is line with our findings of reduced inflammatory cell activation and apoptosis in mice treated with CR2-fH. On the other hand, neuronal growth factor (NGF) and two key mediators of neurogenesis and neuronal migration, VEGF and NCAM, previously reported to exhibit increased expression in migrating neuroblasts [25, 26], were among the significantly upregulated genes.

### CR2-fH increases subacute VEGF levels in the brain after MCAO

**Fig. 8:**
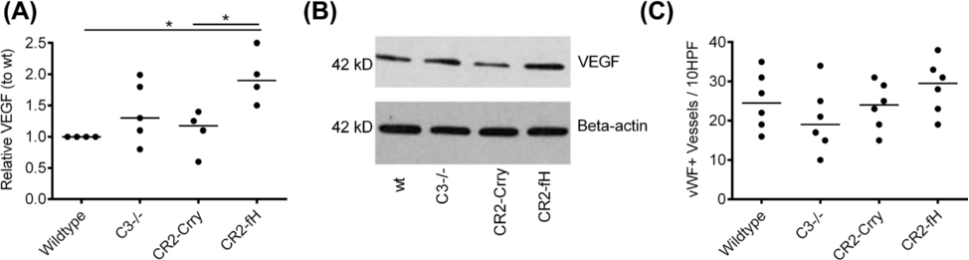
CR2-fH increases relative VEGF expression but not angiogenesis in the ipsilateral brain at 7 days post-MCAO. **a** VEGF expression as determined by Western blot analysis of ipsilateral brain tissue extract. Shown are relative expression levels normalized to level in wt control mice, with β-actin serving as a loading control. *Bar* = Median, *n* = 4, *p < 0.05. **b** Representative Western blot for analysis of VEGF expression. **c** Von Willibrand factor positive (vWF^+^) vessels within the ipsilateral brain as detected by immunohistochemistry. *Bar* = Median (positive vessels per ten high-powered fields), *n* = 6

Since VEGF plays an important role in angiogenesis as well as neuroblast proliferation and migration following ischemia [25], we assessed VEGF levels in the ipsilateral hemisphere 7 days after MCAO. CR2-fH-treated mice had significantly increased levels of VEGF 7 days after MCAO compared to both wt controls and CR2-Crry-treated mice (Fig. 8a, b). Immunostaining for von Willibrand factor (vWF), however, did not reveal any differences in vessel density between the groups at 7 days post-MCAO (Fig. 8c). While it is possible that alternative pathway inhibition with CR2-fH may promote post-ischemia neurogenesis by increasing VEGF levels, it does not appear to have any effect on angiogenesis in the subacute phase. These data support the above Nanostring data and further implicate VEGF in the observed CR2-fH-dependent increase in neuroblast proliferation and migration.

### CR2-fH improves spatial reference memory and avoidance learning in the subacute phase after stroke

**Fig. 9:**
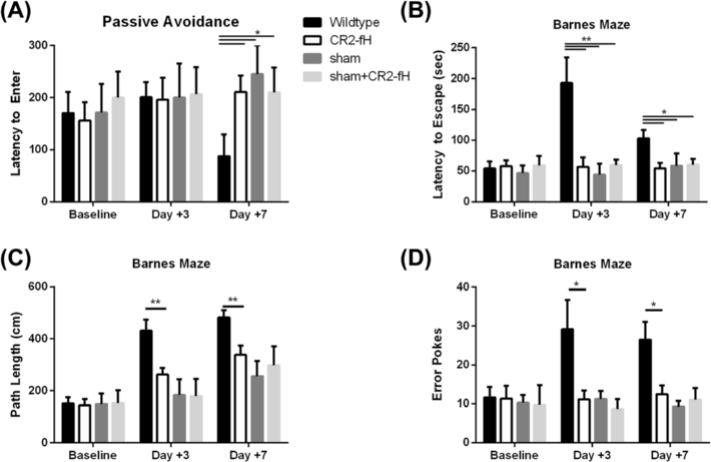
CR2-fH improves cognitive performance 7 days post-MCAO. **a** CR2-fH improves performance on passive avoidance task 7 days after MCAO. Shown is latency to enter a dark box associated with a shock. Mice were given a trial to associate a shock with the dark side of an apparatus, and latency to enter dark side was evaluated on days 3 and 7 post-MCAO. Mean +/− SEM, *n* = 7–10, *p < 0.05. **b**–**d**, CR2-fH reduces spatial memory deficits in Barnes maze task in the subacute phase after MCAO. Mice were trained on the maze for 5 days before surgery and then tested on days 3 and 7 after MCAO for latency to escape **(b)**, path length **(c)**, and number of error pokes **(d)**. Mean +/− SEM, *n* = 9–10, *p < 0.05, **p < 0.01

Based on the significant sparing of hippocampal damage and increased neuroblast migration resulting from CR2-fH treatment, we performed a more detailed investigation of cognitive outcome by analysis of spatial reference memory and avoidance learning using Barnes maze and passive avoidance tasks. On passive avoidance task, there were no significant differences between wt controls and CR2-fH-treated mice 3 days post-MCAO, with mice from both groups recording latency to enter times similar to the times that were recorded pre-surgery (Fig. 9a). However, on day 7 post-MCAO, there was a significant deterioration in performance of the wt control group, whereas the performance of CR2-fH-treated mice was not significantly different to that recorded pre-surgery.

On Barnes Maze task 3 days post-MCAO, the performance of wt control mice deteriorated significantly, as manifested by an increase in latency to escape, path length, and number of error pokes compared to pre-surgical baseline. By day 7 post-MCAO, performance of this group was improved, but was still significantly worse compared to pre-surgical baseline (*p* < 0.05). In contrast, CR2-fH-treated mice displayed no impairment on Barnes maze performance at either 3 or 7 days post-MCAO compared to pre-surgical baseline (Fig. 9b–d). Thus, compared to wt controls, CR2-fH treatment significantly improved spatial reference memory through the subacute phase after ischemic stroke.

To control for possible cognitive-enhancing effects of CR2-fH, we also treated sham-operated mice with CR2-fH and tested cognitive performance after sham surgery on both tasks. CR2-fH treatment did not enhance cognitive performance in sham-operated mice (Fig. 9). The above data together show that a single post-reperfusion dose of CR2-fH not only improves neurological/locomotor and histological measures in the subacute phase after stroke, but also improves cognitive performance.

### CR2-fH treatment reduces neutrophil infiltration into the brain after MCAO

**Fig. 10:**
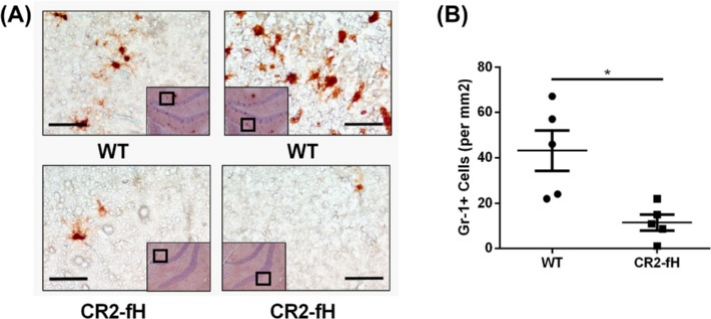
CR2-fH reduces brain neutrophil infiltration at 7 days after MCAO. **a** Representative images of neutrophil infiltration assessed by immunohistochemical staining with anti-Gr-1 antibody. Scale bar = 50 μm. **b** Quantification of Gr-1+ cells using 10 HPF/brain. *Bar* = Mean +/− SEM, *n* = 5, *p < 0.05

Data from Nanostring analysis showed a downregulation in markers of neutrophil activation after MCAO and CR2-fH treatment, and we therefore examined neuronal infiltration by staining sections with the neutrophil marker Gr-1. Compared to wt, CR2-fH treatment resulted in a significant (threefold) reduction in the number of Gr-1-positive cells, suggesting a significant reduction in neutrophil infiltration into the parenchyma (Fig. 10a, b). These data supplement the data above showing a significant reduction in the activation of resident inflammatory cells in the brain after CR2-fH treatment.

### Acutely administered CR2-fH targets to the post-ischemic brain and persists into the subacute phase after stroke

**Fig. 11:**
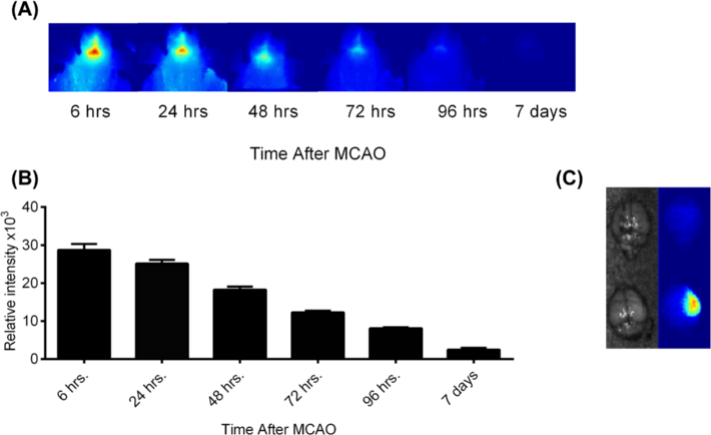
CR2-fH localizes to the brain after MCAO and specifically targets the ipsilateral lesion site. Fluorescently labeled CR2-fH was administered as described for therapeutic protocol, and localization of CR2-fH was visualized by in vivo fluorescence tomography. **a** Representative images of head scans of a single mouse taken at indicated times. **b** Quantification of fluorescent signal. Mean +/− SEM, *n* = 5 (24–72 h), *n* = 4 (72 h), *n* = 3 (7 days). **c**, Ex vivo image of brain removed 7 days after MCAO and injection of labeled CR2-fH (*lower*) or PBS (*upper*)

We have previously demonstrated C3d-specific binding of CR2-fH [15], and to investigate C3d deposition and the targeting and retention of CR2-fH in the post-ischemic brain, we performed in vivo fluorescence imaging. Fluorescently labeled CR2-fH was administered according to our therapeutic protocol, and shaved heads of mice (hair removed to prevent signal interference) were imaged over a 7-day period post-reperfusion. Targeting and localization of CR2-fH was clearly evident 6 h after reperfusion, with a gradual decline to near undetectable levels in live animals by 7 days (Fig. 11a, b). The calculated tissue half-life based on fluorescence signal was 48.5 h. No fluorescence was detected in the brains of sham-operated animals at any time point (not shown). Although fluorescent signal was barely detectable in live animals 7 days after MCAO, ex vivo imaging of the brain removed after sacrifice on day 7 demonstrated the continued presence of labeled CR2-fH, with localization to the ipsilateral parenchyma (Fig. 11c).

### Complement inhibition does not increase post-stroke mortality

**Fig. 12:**
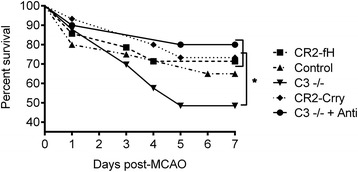
CR2-fH and CR2-Crry treatment but not C3 deficiency improves survival at 7 days post-MCAO. Kaplan-Meier survival analysis of C3-deficient or complement inhibitor-treated mice over a 7-day period post-MCAO. One group of C3-deficient mice received antibiotic prophylaxis (C3 −/− + Anti), *n* = 19–33, *p < 0.05

Immunosuppression and associated complications such as pneumonia are a major concern in stroke patients, and are a frequent cause of morbidity and mortality both clinically [27, 28] and in the experimental model used here [29]. As complement plays an important role in host defense, we determined whether treatment with CR2-Crry or CR2-fH affects animal survival in our model. Neither inhibitor significantly affected 7-day survival compared to control-treated animals (Fig. 12). On the other hand, C3-deficient mice had significantly reduced 7-day survival compared to mice receiving inhibitor treatment. To investigate whether increased mortality in C3-deficient mice may be due to compromised host defense, we treated C3-deficient mice with antibiotics for 2 weeks prior to MCAO and for 7 days post-reperfusion. Antibiotic treatment reversed the effect of C3 deficiency on animal survival, with survival rates the same as for CR2-fH-treated mice. These data indicate that CR2-fH treatment does not compromise complement-dependent host defense mechanisms and increase susceptibility to secondary infectious complications following experimental ischemic stroke.

## Discussion

Complement-mediated inflammation has been shown to play an important role in the progressive degenerative events that take place after ischemic stroke, but there is increasing evidence that complement is also involved in subsequent repair and regenerative mechanisms that occur during recovery [3]. Numerous studies have shown that complement deficiency or inhibition is protective in the acute phase after stroke (24–48 h), but it has also been shown that complement deficiency can worsen subacute outcomes [11, 14]. The reasons for the differences in acute and subacute outcomes are not well understood, but it is clear that complement has a balancing role in injury vs. protection in many pathological conditions, and differences in acute and subacute outcomes after stroke may be due to balancing roles for complement in early inflammation and injury vs. subsequent neuroprotection and neurogenesis. In the context of neuroprotective functions of complement, both C3a and C5a have been shown to be protective against excitotoxic neuronal injury [30–32], and C3a has a protective role in neonatal hypoxia-ischemia brain injury [33], even though C1q exacerbates injury in a similar model [34]. C3a is also implicated in promoting neurogenesis, both basal [14] and following ischemic stroke [34], and similar to C5a, it has been shown to regulate the differentiation and migration of neural progenitor cells in vitro [35, 36]. In addition, complement opsonins of the classical (C1q), lectin (MBL, ficolins), and alternative (properdin) pathways, as well as C3 opsonins common to all pathways, promote the clearance of apoptotic cells and debris, which is important for the resolution of inflammation and recovery [35, 37, 38].

**Fig. 13:**
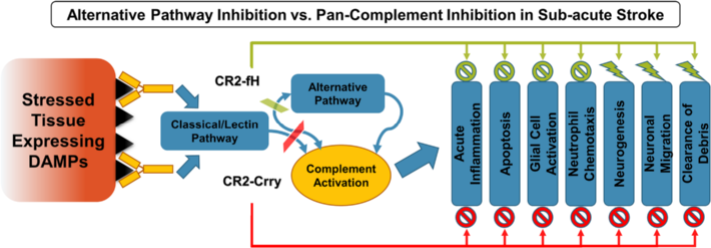
Schematic depicting complement activation and the effects of CR2-Crry vs. CR2-fH on complement-dependent effector mechanisms after ischemic stroke

The data presented here support the notion of a dual role for complement in ischemic stroke by comparing C3 deficiency with transient approaches of complement inhibition using CR2-Crry and CR2-fH (Fig. 13). We demonstrate that whereas C3 deficiency and complement inhibition provides acute protection after ischemic stroke, only complement inhibition reduces inflammation and cell death in the subacute phase, and only inhibition of the alternative pathway with CR2-fH prevents evolution and spread of injury. The transient nature of complement inhibition provides an explanation for the worse subacute outcomes in complement-deficient vs. complement-inhibited mice. Complement deficiency will prevent the early pro-apoptotic and pro-inflammatory effects of complement mediated by the anaphylatoxins and opsonins that trigger complement-dependent phagocytosis, an effect that is also achieved by complement inhibition. However, complement inhibition will allow for recovery of complement function at the subacute phase permitting for critical neuro-reparatory functions of the complement system that involve promoting neurogenesis, clearance of debris and resolution of inflammation. To support this hypothesis, we analyzed the temporal profile of CR2-fH in the brain after a single injection and demonstrated targeted post-ischemic binding with diminishing levels during progression to the subacute phase, which would presumably allow complement-mediated homeostatic and neuroprotective mechanisms to become operable as inflammation is resolved.

The use of two different complement inhibitors in this study allowed us to investigate the selective contribution of the alternative complement pathway in secondary injury after stroke. Of the two complement inhibitors investigated, only CR2-fH improved neurological deficit and locomotor function at 7 days after stroke. Improved motor outcome at 7 days in CR2-fH-treated mice compared to CR2-Crry-treated mice and C3-deficient mice is associated with significantly lower cortical cell death in the CR2-fH-treated group. In addition, only CR2-fH-treated mice had significantly reduced hippocampal cell death, and only CR2-fH-treated mice did not display a reduction in neurogenesis markers in the SVZ. In addition to maintaining post-stroke neurogenesis, CR2-fH treatment also promoted significantly more neuroblast migration from the SVG compared to wt mice, findings that correlated with improved performance on spatial learning and passive avoidance tasks in the subacute phase after stroke. Since complement activation products are implicated in acute injury as well as recovery and neurogenesis after CNS injury, a mechanism accounting for the different subacute outcomes in CR2-fH- vs. CR2-Crry-treated mice may be related to a difference in the extent of complement activation. Current evidence indicates that following cerebral ischemia and reperfusion, complement is activated by the lectin pathway [5–9] and amplified by the alternative pathway [4]. As outlined above, C3a and C5a have neuroprotective/regenerative roles, and whereas CR2-fH treatment significantly inhibited C5a generation in the ipsilateral brain for up to 3 days after stroke, C5a levels were still significantly higher than in brains from CR2-Crry-treated mice. Thus, CR2-fH maintains a baseline level of C5a generation through the subacute phase that correlates with improved overall post-stroke recovery, and based on previous data, this likely occurs via the lectin pathway. Differences in the extent of complement activation could also account for our observations that CR2-Crry is more effective than CR2-fH at reducing acute injury (lesion size), even though longer-term outcomes are worse. The concept of CR2-fH providing optimal longer-term protection by self-limited complement activation is further supported by findings that high-dose, but not low-dose, C3aR antagonism impaired post-stroke SVZ neurogenesis in the subacute phase [14, 24]. CR2-fH, but not CR2-Crry, also increased VEGF expression within the ipsilateral hemisphere, and this may point to another protective mechanism of CR2-fH. Although the role of complement in post-stroke angiogenesis has not been investigated, C3a and C5a have been shown to promote VEGF expression and pathogenic neovascularization within the retina in a model of age-related macular degeneration [39]. We did not observe differences in angiogenesis with complement inhibition, but it is possible that this may occur at later time points.

Regarding post-stroke neurogenesis, findings are mixed on whether newly forming neurons make a functional impact on plasticity and recovery. Here, we investigated neurogenesis and neuroblast migration 7 days following ischemic stroke, and show that increased neuroblast migration to the basal ganglia and hippocampal area in CR2-fH-treated mice is indeed associated with improved performance on cognitive and memory tasks. The effect of CR2-fH on neurogenesis and neuroblast migration was also associated with increased levels of NCAM and VEGF transcript and VEGF protein. Both molecules have been studied in the context of neurodevelopment, with VEGF expression shown to induce neuroblast proliferation and migration, while NCAM is a neuronal adhesion molecule essential for the formation of chains of migrating neuroblasts during development [26] It is noteworthy that CR2 expression in neural progenitor cells has been shown to regulate hippocampal neurogenesis, and treatment with the CR2 ligand, C3d, was shown to reduce the number of proliferating neuroblasts in vivo [40]. It is therefore possible that the CR2 targeting moiety of CR2-fH could be contributing to its function by competing with the interaction of C3d with CR2 expressed on neural progenitor cells, although it is unlikely this represents a principle mechanism of action since there is inhibition of neurogenesis in CR2-Crry-treated mice compared to wt.

The C3a and C5a anaphylatoxins are implicated in promoting immune cell migration and infiltration, and may also be involved in neuroblast migration. Interestingly, however, while we show that the significant reduction of C5a levels in the brains of CR2-fH-treated mice is associated with decreased immune cell infiltration (especially neutrophils), it is associated with enhanced neuroblast migration. CR2-fH inhibited but did not completely block C5a generation, and there may be different thresholds for anaphylatoxin stimulation or different mechanisms of action of the anaphylatoxins in immune cell vs. neuroblast migration, and these data again highlight the dual role of complement in injury and repair.

Additional differences between C3-deficient and complement-inhibited mice were the extent of microglial activation and post-stroke mortality. C3-deficient mice had significantly higher activation of microglial/macrophage cells compared to complement-inhibited mice. The reason for the higher levels of microglial/macrophage activation in C3-deficient mice is not clear, but may be related to the increased levels of striatal apoptosis found in complement-deficient vs. inhibited mice. In this regard, C3 opsonization of apoptotic cells is known to play a role in apoptotic cell clearance, and the complete absence of this process in C3-deficient mice may more severely restrict cell clearance and impair the resolution of inflammation. In addition, C3-deficient mice had a significantly higher mortality rate compared to complement-inhibited mice. Treatment of C3-deficient mice with an antibiotic eliminated this difference, indicating that increased mortality was due to infection. Complement plays an important role in host defense mechanisms, and our finding here is also in accord with a previous study in which it was shown that C3 deficiency, but not CR2-Crry treatment, increased susceptibility to infection in a model of septic peritonitis [16]. CR2-mediated targeting obviates the need for systemic complement inhibition [15, 16], and the current data indicate that CR2-fH does not increase susceptibility to infection following experimental ischemic stroke. Secondary infectious complications are a serious concern for stroke patients, and infection (septicemia and pneumonia) is a major cause of death following stroke in the model we use here [29]. Other known causes of mortality after stroke include hemorrhagic transformation, brain edema and, with regard to experimental stroke, loss of mobility leading to malnutrition and dehydration of the animal.

## Conclusions

In conclusion, we show that an acutely administered dose of CR2-fH reduced injury and improved neurological and behavioral outcomes in the subacute phase after stroke. These improved outcomes were linked to reduced extent of cell death and increased neurogenesis and VEGF expression. The improved longer-term outcomes in CR2-fH-treated mice compared to CR2-Crry-treated and C3-deficient mice, and the inhibition of neurogenesis in CR2-Crry-treated and C3-deficient mice, indicate that CR2-fH provides targeted and self-limiting complement inhibition that dissects the dual role of complement in injury and recovery after stroke. This conclusion is supported by data showing that CR2-Crry more completely blocks complement activation than CR2-fH, as determined by comparing post-stroke brain levels of the complement activation product, C5a. This approach of alternative pathway inhibition offers potential advantages over systemic and/or regulated doses of complement inhibitors that may be contraindicative to long-term outcome. The current findings also warrant further investigation into therapeutically relevant issues such post-stroke therapeutic window, outcomes in older mice with increased stroke risk factors or with pre-existing cerebrovascular disease, and outcomes in pre- and post-menopausal female mice.

## Additional file

Additional file 1:
**List of 248 genes and results of Nanostring mRNA expression analysis comparing wild-type control to CR2-fH-treated mice.**
